# Versatile properties of dynein molecules underlying regulation in flagellar oscillation

**DOI:** 10.1038/s41598-023-37242-6

**Published:** 2023-06-29

**Authors:** Takashi Fujiwara, Chikako Shingyoji, Hideo Higuchi

**Affiliations:** 1 Department of Biological Sciences, Graduate School of Science, The University of Tokyo, Tokyo, Japan; 2 Department of Physics, Graduate School of Science, The University of Tokyo, Tokyo, Japan; 3 Universal Biology Institute, Graduate School of Science, The University of Tokyo, Tokyo, Japan

**Keywords:** Biophysics, Cell biology

## Abstract

Dynein is a minus-end-directed motor that generates oscillatory motion in eukaryotic flagella. Cyclic beating, which is the most significant feature of a flagellum, occurs by sliding spatiotemporal regulation by dynein along microtubules. To elucidate oscillation generated by dynein in flagellar beating, we examined its mechanochemical properties under three different axonemal dissection stages. By starting from the intact 9 + 2 structure, we reduced the number of interacting doublets and determined three parameters, namely, the duty ratio, dwell time and step size, of the generated oscillatory forces at each stage. Intact dynein molecules in the axoneme, doublet bundle and single doublet were used to measure the force with optical tweezers. The mean forces per dynein determined under three axonemal conditions were smaller than the previously reported stall forces of axonemal dynein; this phenomenon suggests that the duty ratio is lower than previously thought. This possibility was further confirmed by an in vitro motility assay with purified dynein. The dwell time and step size estimated from the measured force were similar. The similarity in these parameters suggests that the essential properties of dynein oscillation are inherent to the molecule and independent of the axonemal architecture, composing the functional basis of flagellar beating.

## Introduction

The cyclic beating of eukaryotic flagella originates from oscillating internal components, forming a “9 + 2 structure” that consists of various components, including nine dynein-bound doublets and paired microtubules in the center. The motive force for beating is produced by dynein-driven microtubule sliding, in which every neighboring doublet microtubule in the axoneme slides past each other^1–3^. The bending produced at the base of a flagellum is sequentially propagated to the tip. The sliding between doublet microtubules and dynein molecules is first experimentally demonstrated using trypsin-treated axonemes^4,5^. Later, axonemes disintegrate into individual doublets with trypsin or elastase in the presence of adenosine triphosphate (ATP) (0.01–1 mM)^4,6,7^. With such materials, all dynein molecules might be the generator that invokes the sliding force at every part along the flagellum. Combined with structural and in vitro motility studies, dynein produces force toward the plus-ends of microtubules^6,8–10^. Since all nine doublets begin as the minus-end at the base of flagellum, cyclic beating and its propagation might be the consequence of the cooperative sliding among doublet microtubules, occurring at the adequate location of the axoneme with proper timing^11^. Regular sliding would be achieved by an orchestrated feedback system in response to the precise mechanochemical switch as an intrinsic feature of dynein molecules^12,13^.

Although the detailed mechanism of flagellar oscillation that regulates microtubule sliding remains a mystery, analyses of individual or various combinations of axonemal components suggest some attractive ideas to solve the missing links, even partially. Among these components, central-pair microtubules have recently been focused on as crucial parts because of their potential roles in determining the bending planes of the flagella^14–17^. The contribution of the central pair is determined by a comparison between the force generation of axonemal structures in the presence or absence of central-pair microtubules.

By measuring the force exerted by dynein on doublet microtubules with optical tweezers, the force by a small number of dynein molecules certainly generates oscillation^18^. However, the properties of the force generated under more complicated states near intact flagella remain unclarified. This strongly prompts careful characterization of the differences among systems with different constituents. Shingyoji and others^19^ have confirmed that trypsin induces excessive fragmentation of outer arm dyneins; milder digestion with elastase partially disintegrates the axoneme into thick and thin bundles that include intact dyneins and fewer doublets^20–23^. The effective use of this treatment enables us to examine the sliding properties of partially disintegrated axonemes under near-physiological states, especially those in the presence or absence of central-pair microtubules. In this study, we compared the properties of the force generated by dynein at three dissection stages of axonemes to evaluate the remarkable performance of a small number of dynein molecules, anticipating the presence of some significant differences in the mode of force-generation among them.

## Results

### Three dissection stages reflecting the dynein generation of force in axonemes

**Figure 1:**
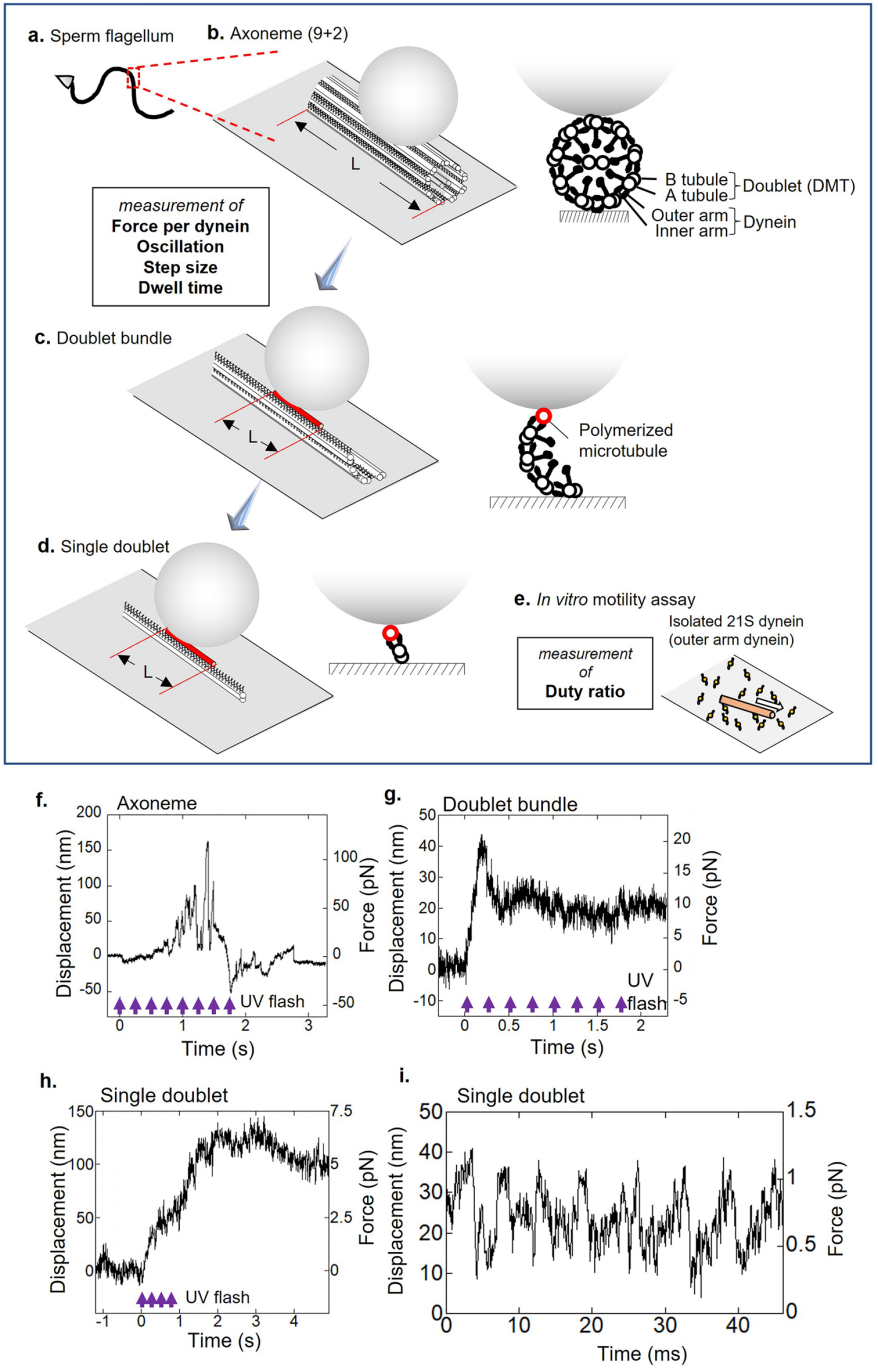
Schematic drawings of the experimental design (**a–e**) and the typical force generation of substructures of axonemes (**f**–**i**). (**a**) Sperm flagellum of the sea urchin, used as a material, has a “9 + 2 structure”. (**b**) Force measurements on “Axonemes”. The bead was brought into contact with doublets of “Axoneme” via inactive kinesin (see methods) as a handle for optical tweezers. (**c**) Dynein force measurements on “Doublet bundles” without a central pair. The bead was manipulated to interact with a polymerized microtubule (red) via avidin on the bead and biotin on the microtubule that interacted with dynein molecules on the doublet. The length of the microtubule was defined as L. (**d**) For the force measurements of dynein on “Single doublets”, a bead was attached to microtubules by the same method as "Doublet bundles". (**e**) In vitro assay in which isolated 21S dynein molecules on a glass surface translocated a microtubule (orange). (**f**–**h**) Typical time courses of force generation measured from (**f**) “Axoneme”, (**g**) “Doublet bundle”, and (**h**) “Single doublet” specimens. For "Axonemes” and “Doublet bundles”, to maintain high ATP concentrations, 4 or 8 times the series of photolysis of caged-ATP by UV flashes with an interval of 250 ms was applied. Purple arrows below the displacement show repetitive UV flashes. (**i**) Example of oscillatory force generated by “Single doublet”.

We aim to understand the basis of force generation by dynein that causes oscillation in flagella (Fig. 1a–e). To deeply understand the oscillation mechanism, it is essential to recognize whether structural dissection causes any defect or changes in dynein activities. The dissection of axonemal structures that retain beating functions is particularly important. For dynein force measurement, we introduced sophisticated methods utilizing dynein in three substructures of the axoneme: dynein on doublets within a fragmented axoneme (Fig. 1b), dynein on a doublet at the edge of a bundle of 3–6 doublets (Fig. 1c) and dynein on a single doublet disintegrated from a fragment of the axoneme (Fig. 1d). In this work, we simply denote three structures as “Axoneme”, “Doublet bundle” and “Single doublet”.

The basic cytoskeletal structure of the flagellar axoneme consists of nine doublet microtubules and a central pair at the center. Intact axonemes were fragmented by a homogenizer to obtain “Axoneme”. An optically trapped bead was attached on “Axoneme” to measure the force of dynein (Fig. 1b). Elastase-digested axoneme separates into several groups (or bundles) of doublets at high ATP and Ca^2+^ concentrations. These responses are likely to be induced by alternating dynein activities on the doublets at both sides of the central pair^15,24^. For dynein on “Doublet bundle”, the elastase-digested “Axoneme” separates into a portion (3–6) of 9 doublets that sustains the original arrangement of axoneme, including the regulating components, such as radial spokes and the nexin-dynein regulatory complex, except for the central pair. At low Ca^2+^ and ATP concentrations, “Axoneme” disintegrates into individual doublets. We confirmed whether the studied samples disintegrate into individual doublets (see the “Materials and Methods” for details). For dynein on “Single doublet” (Fig. 1d), dynein on the doublet retains its sliding ability; however, the central pair is lost, and the function of regulatory components, such as radial spokes and the nexin-dynein regulatory complex, is lost or changed by attaching to the glass surface or detaching from doublet microtubules. Polymerized microtubules interacted parallel to the row of dyneins on the edge of “Doublet bundle” or on “Single doublet”, and then the optically trapped beads were attached to microtubules (Fig. 1c). Dynein force generation was induced by the application of UV photolysis of caged ATP (Fig. 1b–d). All experiments were completed within 30 min after disintegration checking.

Time-dependent changes in dynein-generated forces were measured with optical tweezers under three dissected conditions (Fig. 1f–h). The timing of dynein activation was controlled by the application of UV photolysis of caged ATP (shown by arrows). Despite the technical difficulty in measuring force on axonemal samples, we succeeded in collecting force data up to several dozens of cases. Tracings show up-and-down changes in force, including the rapid elevation and reduction in force (Fig. 1f) or an initial rapid force elevation followed by a slight decrease; this phenomenon maintained the force for over a few seconds after ATP application (Fig. 1g). The others slightly decreased the force (Fig. 1h). The percentages of occurrences of force generation by ATP application were 39% (51 out of total sample number, 131) on “Axoneme”, 37% (23 out of 63) on “Doublet bundle”, and 33% (38 out of 116) on “Single doublet”. Time-dependent changes in force were not largely different among the three conditions. Notably, the basic properties of dynein were found to be similar in these three stages regardless of the structurally large dissection among them.

In addition to the abovementioned type of force generation, all three types of dynein force in dissected conditions oscillated. One example of this force oscillation is shown in Fig. 1i. The frequency measured from the power spectrum density was 156 Hz (Fig. 1i). We present the details of the oscillation features observed later in Figs. 4, 5, 6 and 7.

### Effects of the microtubule or doublet microtubule length on the generation of dynein force

Single molecule of 22S dynein purified from *Tetrahymena* cilia generated a force of ~ 5 pN at a low ATP concentration of 3 µM, while it did not generate force distinguishable from noise at a high ATP concentration of > 20 µM^25^. The question is how single dynein contributes force generation in dynein assembly on three dissected conditions at high ATP concentrations. Since dynein molecules are present periodically along doublet microtubules, the number of dyneins is almost proportional to its length. In this study, therefore, we measured the effects of microtubule/doublet-microtubule length on the dynein force.

**Figure 2:**
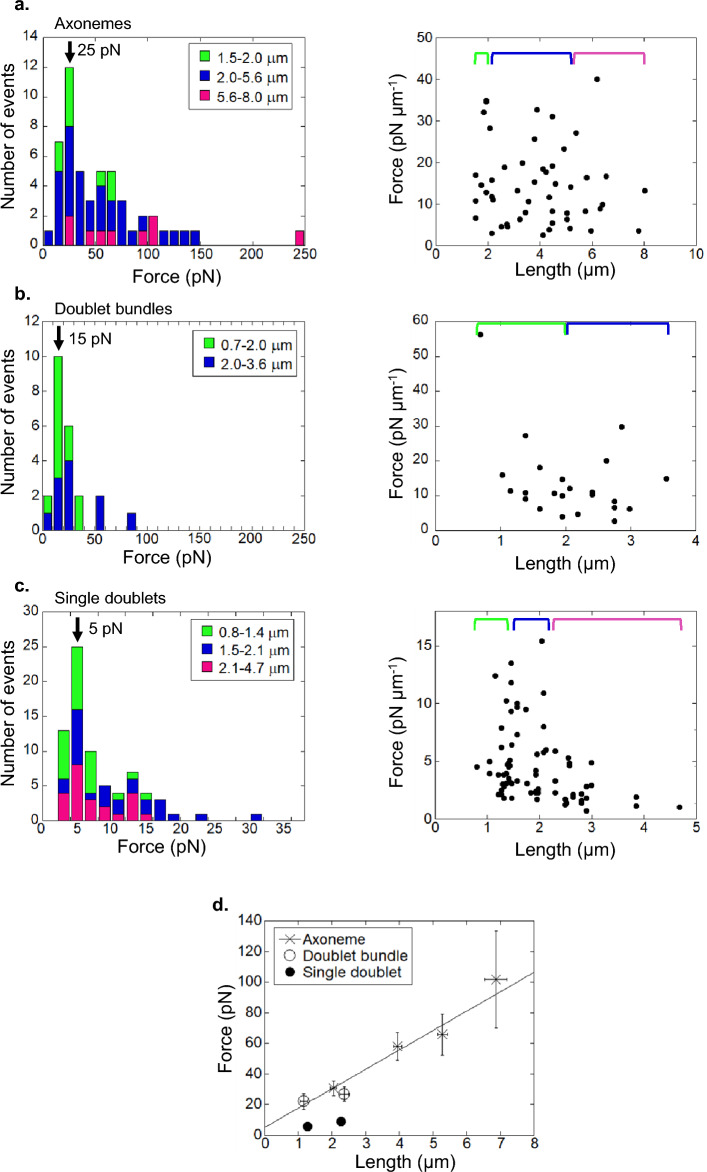
Effects of length changes in microtubules and doublet microtubules on the generation of dynein force. Force distribution of (**a**) “Axonemes” (n = 51), (**b**) “Doublet bundles” (n = 23) and (**c**) “Single doublets” (n = 74 from 38 doublets). The green, blue and magenta bars indicate the ranges of lengths of “Axonemes” (**a**) or interacting microtubules (**b**, **c**). Dynein force tended to increase with an increase in the length of the interacting microtubule. The distribution of each force per unit length is shown in the right columns of **a**–**c**. (**d**) The force measured from dissected conditions was presented as mean values ± s.e. The regression line was fitted to data from “Axoneme” with correlation coefficient of 0.986. Slope: 12.7 ± 3.3 pN µm^−1^ and y-interception: 5.0 ± 10.0 pN.

Before the activation of dynein, we measured the length (L) values of axonemal fragments (Fig. 1b) and polymerized microtubules interacting with the doublet microtubules of “Doublet bundles” (Fig. 1c) or with “Single doublets” (Fig. 1d). After the activation of dynein, the maximum forces generated by dyneins of three conditions were measured. The forces generated by dyneins on both “Axonemes” and “Doublet bundles” increased with increases in the lengths of “Axonemes” and microtubules. While the calculated forces per unit length of “Axonemes” and microtubules interacting with “Doublet bundles” were scattered, they were almost independent of their lengths (Fig. 2a,b). This finding was confirmed by the result in which the forces of “Axonemes” were proportional to their lengths with slopes of 12.7 ± 3.3 pN μm^−1^ (mean ± s.e.) (Fig. 2d). The forces of “Doublet bundles” overlapped with the regression line of the force–length relationship of “Axonemes”. In contrast, the forces of “Single doublets” were much less than those of “Axonemes” and less dependent of the interaction length (5.7 ± 0.6 and 9.1 ± 0.9 pN at length of 1.3 and 2.3 μm, respectively) (Fig. 2d). The predominant force in the histogram was ~ 5 pN independent of the length (Fig. 2c). The small force of “Single doublet” is explained by the suggestion that dynein on short segments of the curved and twisted doublets interacts with microtubules, as discussed later. The force per length of the segment in “Single doublet” is possibly the same (12.7 ± 3.3 pN μm^−1^) as that of “Axoneme” and “Doublet bundle”. The mean force per dynein molecule was calculated to be 0.11 pN (12.7/115) from the force per unit length divided by the number of dyneins (~ 115 μm^−1^)^26,27^. The mean force of dynein on three conditions was only 2–3% of the stall force of a single axonemal dynein (~ 5 pN)^25^. There are two possible reasons for the small mean forces. First, a small fraction of dynein molecules generates different forces simultaneously; second, each dynein molecule on the doublet generates a small force.

### Duty ratio and dynein affinity to microtubules

**Figure 3:**
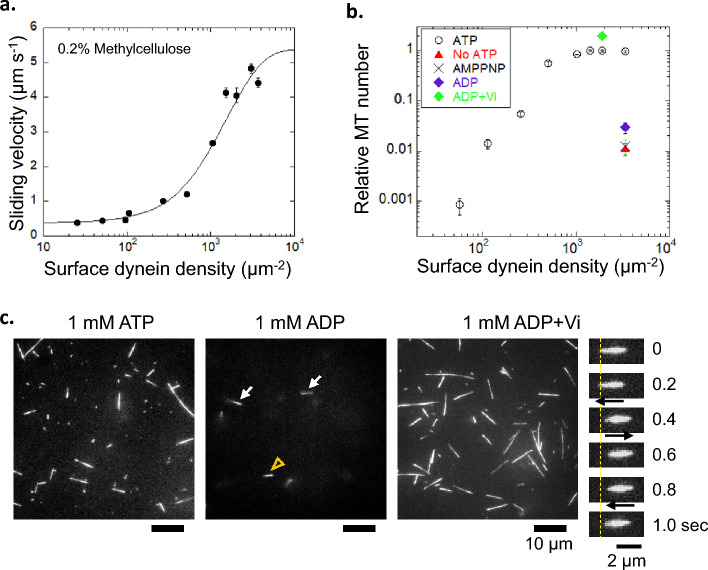
In vitro assay of purified 21S dynein. (**a**) Relationship between the dynein density and sliding velocity of microtubules. The sliding velocity was obtained from 21–46 microtubules on each dynein density. The plots were fitted by the equation *V* = *V*_max_(1 − e^−*Aρf*^)/(1 − e^−*Aρ*^), where* V*_max_, *ρ*, *A* and *f* denote the maximum sliding velocity, surface dynein density, area of dynein interacting with microtubules (length, 4 μm), and duty ratio, respectively. (**b**) Relationship between the surface dynein density and number of microtubules attached to surface dynein in the presence of 1 mM ATP (open circles, n = 10–45), in the absence of nucleotide (no ATP; red rectangle, n = 20), and in the presence of 1 mM AMPPNP (cross, n = 20), 1 mM ADP (purple diamond, n = 20) and 1 mM ADP and 200 µM Vi (green diamond, n = 20). The number of microtubules (length, 2–6 μm) that attached to surface dyneins for approximately 1 s was counted. (**c**) Fluorescence images of microtubules attached to surface dynein on the glass surface. The arrowhead and arrows at 1 mM ADP show microtubules attached for longer and shorter times, respectively, than 1 s. Some microtubules showed Brownian motion at 1 mM ADP and 200 μM Vi. An example of sequential motions is shown in the rightmost panels in (**c**).

To estimate which of the above reasons is the most likely, the duty ratio of purified 21S dynein was investigated using an in vitro gliding assay (Fig. 1e). In this study, the duty ratio was defined as the fraction of time that the motor domain of dynein remained bound to microtubules^28^. Methylcellulose was added to the assay buffer to prevent microtubules from detaching and diffusing from the glass surface when the surface motor density was low^29^. The microtubule gliding velocity of 21S dynein in an in vitro assay increased sigmoidally with an increase in the density of dynein (Fig. 3a). The velocity (*V*) was fitted with the equation of velocity at the surface density (*ρ*) of 21S dynein to obtain the duty ratio (*f*):

1 $$V = V_{max} \left( {1 - e^{ - A\rho f} } \right)/\left( {1 - e^{ - A\rho } } \right),$$

where *V*_*max*_ and *A* are the maximum velocity and the area where 21S dynein reaches microtubules, respectively^30^. The duty ratio was 0.069 ± 0.004, and *V*_*max*_ was 5.4 ± 0.2 μm s^−1^ (mean ± s.e.). The low duty ratio of ~ 7% supports the idea that a small fraction of the axonemal dynein generates force simultaneously.

To understand the reason for the low duty ratio of 21S dynein, we evaluated the affinity of 21S dynein to microtubules at several intermediate states of dynein ATPase without methylcellulose. The number of microtubules interacting with dynein on the glass surface for a duration of ~ 1 s was counted in the presence of 1 mM ATP under various surface densities of dynein as standard conditions (Fig. 3b,c). The number of attached microtubules increased sigmoidally with increasing dynein density and then became constant at a dynein density of > 1000 μm^−2^. In the presence of AMPPNP and ADP and in the absence of nucleotides, few microtubules were bound to dynein on the glass surface (Fig. 3b,c). Under these conditions, the number of binding microtubules relative to that at 1 mM ATP was 0.01–0.03 at ~ 3000 μm^-2^. The small relative number (0.01–0.03) corresponded to a dynein density of ~ 100 μm^−2^ in the case of 1 mM ATP (Fig. 3b). This result indicates that the MD-AMPPNP, MD-ADP, and MD-apo states have ~ 1/30 times lower affinities than those in the presence of 1 mM ATP.

The number of microtubules bound to dynein in the presence of vanadate and ADP was twice that in the presence of 1 mM ATP (Fig. 3b,c). We found that some microtubules showed Brownian motion along their long axes (Fig. 3c, right). The translational diffusion coefficient along microtubules was calculated as 3.3 × 10^4^ and 1.3 × 10^4^ nm^2^ s^−1^ for mean microtubule lengths of 1.6 and 3.6 µm from the slope of the relationship between the time and the mean square distance (Supplemental Fig. S1). The diffusion coefficients of microtubules interacting with dynein in the ADP-Vi state were approximately 60 times smaller than those calculated in the solvent. This finding indicates that dynein binds weakly to microtubules, suppressing microtubule diffusion by weak binding. Based on the idea that dynein-ADP-Vi and dynein-AMPPNP states mimick the dynein-ADP-Pi and dynein-ATP states, respectively, dynein affinity to microtubules was not high in the ATP, ADP-Pi, ADP and apo states. These results suggest that the low affinity of dynein to microtubules is coupled to the low duty ratio of 21S dynein.

### Generation of force-oscillation

**Figure 4:**
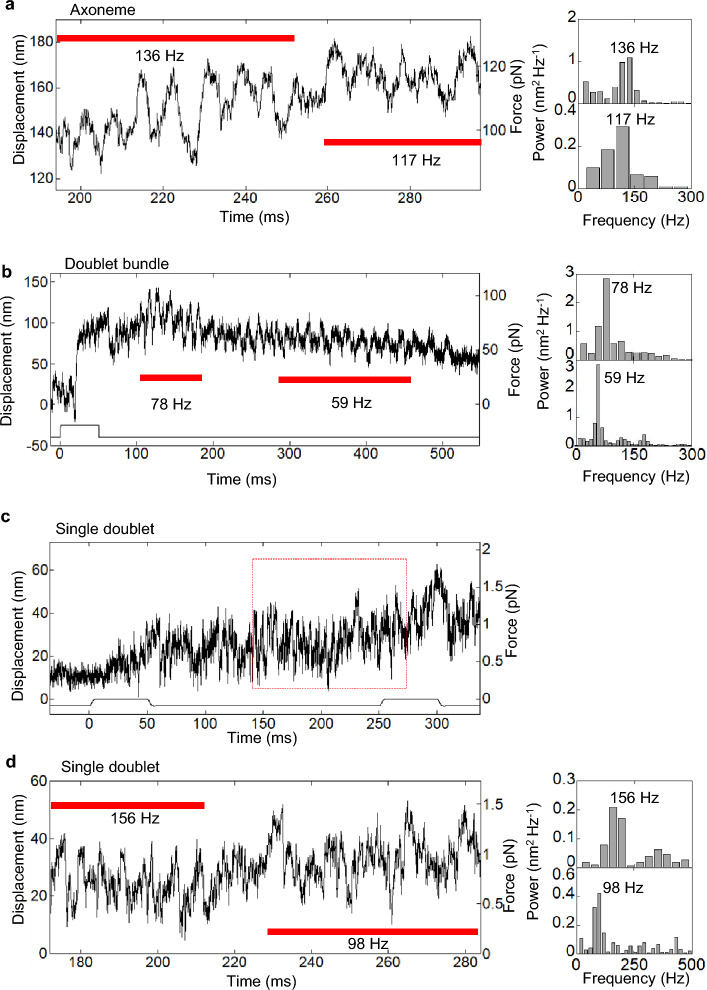
Overview of oscillatory force generation (left) and corresponding power spectra for the regions shown by red bars (right). (**a**), “Axoneme”, (**b**) “Doublet bundle” and (**c**, **d**) “Single doublet”. (**b**) Oscillation of “Doublet bundle” was obtained after disintegration of the bundle. (**d**) is a magnified trace of the region shown by a broken rectangle in (**c**). The time zero was set at the initiation of the UV flash. The peak frequencies are indicated on each power spectrum.

In this work, we define the “oscillation” from the displacement-tracings according to the criteria that satisfy four requirements; (1) the amplitude of the main frequency in the power spectrum is higher than twice that of the rest, (2) the waves repeat more than 4 times, (3) the peak-to-peak amplitudes of the waves are larger than 8 nm and (4) the waves start within 2 s after triggering UV flash (detail, see Materials and methods). Previous reports showed that full length axoneme and single doublet interacted with microtubule at angle of 45–90° certainly generate oscillatory forces^18,31,32^. Here, we observed, for the first time, the oscillations in the force generated by dynein under three architectural conditions. Number of oscillation samples among force generating samples were two among 51 samples for “Axonemes” (Fig. 4a), one among 23 samples for “Doublet bundles” without disintegration, one among two samples for “Doublet bundles” with disintegration (Fig. 4b) and seven among 38 samples for “Single doublets” (Fig. 4c,d). Oscillations were sometimes observed a few times in each sample. The total numbers of oscillation observed were 3, 3 and 22 for “Axonemes”, “Doublet bundles” and “Single doublets”, respectively.

**Figure 5:**
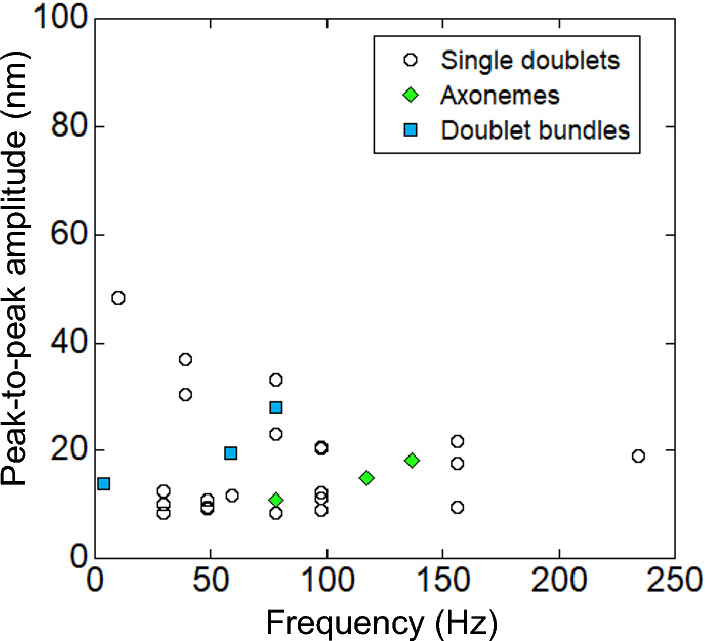
Relationship between the frequency and the amplitude of oscillatory force. The open circles are data obtained from “Single doublets” (n = 22). Oscillatory forces generated by “Axonemes” (green diamonds) and “Doublet bundles” (blue rectangles) are shown.

Predominant frequencies of the oscillations were obtained from the largest peaks of power spectrum densities (right figures in Fig. 4). The peak-to-peak amplitudes were calculated from the power spectrum density and the width of the peak. The frequency and amplitudes of oscillations of “Single doublets” were scattered very much from 10 to 234 Hz and from 8.3 to 48 nm, respectively (Fig. 5, open circles). The amplitude tended to decrease with the increase in frequency. The data points of “Axonemes” (3 data) and “Doublet bundles” (3 data) (Fig. 5, green diamonds and blue rectangles, respectively) were within the frequency and amplitude of “Single doublets”, indicating that the wave-characteristics of all three substructures resemble each other.

**Figure 6:**
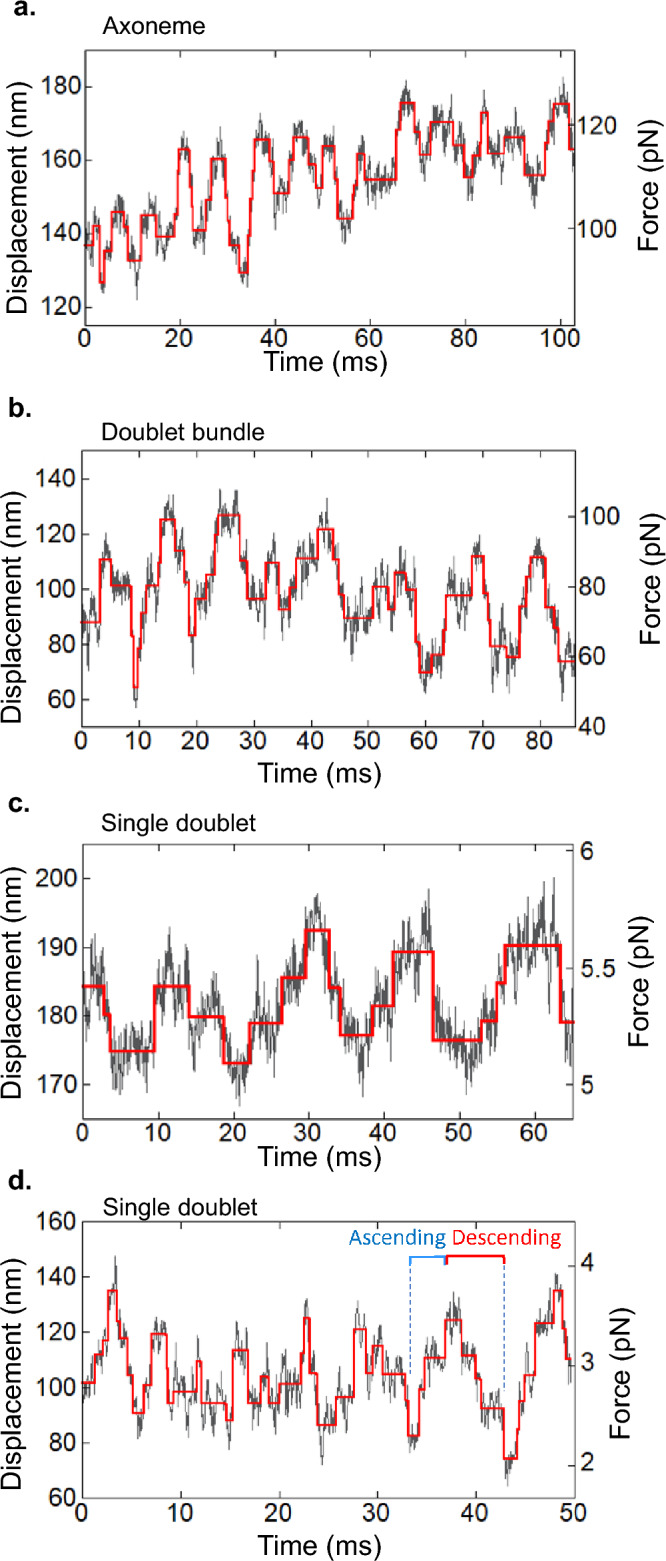
Oscillation by dynein on doublet microtubules in three preparations. (**a**) “Axoneme”, (**b**) “Doublet bundle” and (**c**, **d**) “Single doublets”. The displacements (gray) are found without processing the low-pass filter and the steps are found by the step-finding algorithm (red). The ascending and descending phases are indicated in (**d**).

**Figure 7:**
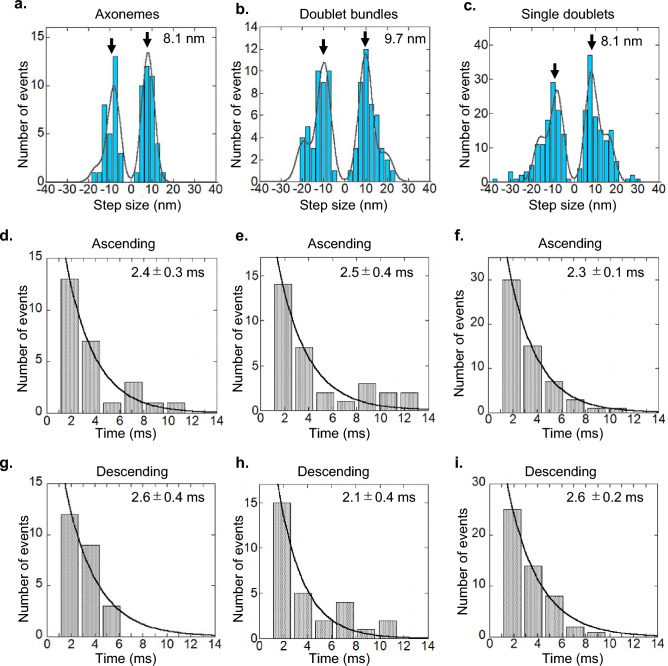
Analyses of step size and dwell time values on oscillation. The distributions of step sizes of (**a**) “Axonemes” (n = 2; mean frequency of 98 Hz), (**b**) “Doublet bundles” (n = 2; mean frequency of 69 Hz) and (**c**) “Single doublets” (n = 8; mean frequency of 66 Hz). The distributions were fitted to multiple Gaussian functions with integer multiples (−2, −1, 1, 2) of step size. The numbers beside arrows show the peak values of the Gaussian function. (**d**–**i**) Distributions of dwell times in the ascending phase (**d**–**f**) and the descending phase (**g**–**i**). (**d**, **g**) “Axonemes”, (**e**, **h**) “Doublet bundles”, and (**f**, **i**) “Single doublets”. The dwell times were fitted to single exponential functions (gray). The obtained time constants are shown in figure (**d**–**i**).

Stepwise displacements of microtubule driven by dynein molecules on oscillatory force generations were found by a step-finding algorithm^33^ (Fig. 6). Most of oscillation had a few steps in ascending and descending phases of oscillatory waveform. The distributions of step sizes were fitted well with multiple Gaussian function with integer multiple of 8.1 ± 0.3, 9.7 ± 0.3, and 8.1 ± 0.2 nm (mean ± s.e.) for “Axonemes”, “Doublet bundles” and “Single doublets”, respectively (Fig. 7a–c). The dwell times of sequential steps (two or more steps) in ascending or descending phases (Fig. 6d) were analyzed. The distributions of the dwell times were well-fitted by single exponential curves (Fig. 7d–i). The dwell times (2.3–2.5 ms, Fig. 7d–f) in ascending phases of oscillations did not differ significantly from those in the descending ones (2.1–2.6 ms, Fig. 7g–i) for all preparations. The step size and dwell times of “Single doublets” were not significantly different from those of “Axonemes” and “Doublet bundles” (Fig. 7d–i). These observations suggest that the essential characteristics, i.e., amplitude-frequency relationship, step size and dwell times, of oscillation are common among “Axonemes”, “Doublet bundles” and “Single doublets”, and probably derive from the simplest last architecture, i.e., single or small numbers of dynein molecules^18^.

## Discussion

To elucidate the mechanochemical characteristics of dynein molecules in axonemes, we measured the force and oscillation generated by dynein molecules under three dissection conditions; “Axoneme”, “Doublet bundle” and “Single doublet”, and measured the dwell time, step size and duty ratio in each system. This is the first attempt which nobody could achieve so far, because of the difficulty of both axonemal dissection and force measurement from the axonemal samples. Resultantly, it was found that the force generated by dynein oscillates under all those conditions. The forces developed by dynein molecules for all preparations were low, ~ 13 pN per micrometer (“Axoneme” and “Doublet bundle”) or ~ 8 pN (mean force of “Single doublet” at length of 1.3 and 2.3 μm). This was consistent with the low duty ratio of purified dynein. The dynein on the doublet generated oscillatory motion with a step of ~ 8 nm. We hypothesized that dissecting axonemal samples into simpler structures, specifically by removing regulatory components such as the central pair and radial spokes through axonemal dissection, would result in change in oscillatiory characteristics. However, the characteristic parameters of oscillation were almost common among all preparations. Such mechanochemical properties shared among three structurally different preparations suggest that flagellar beating is coupled with the oscillation of individual or very few number of dynein molecules.

### Force generation by a small fraction of dynein

The duty ratio is a key characteristic of the mechanochemical reactions of motor proteins to determine the force of the motor ensemble^28^. The forces exerted by the short fragments (2–8 μm) of “Axonemes” were 12.7 ± 3.3 pN μm^−1^ (Fig. 2d). These forces were not significantly different from those (28 ± 11 pN μm^−1^) of full-length axonemes (~ 40 μm in length) measured by glass needles at high ATP concentrations^34^. The force per length of “Doublet bundles” without a central pair was not significantly different from that of “Axonemes” with a central pair. Our results contradict previous reports that suggest the necessity of regulating dynein activity by the central pair and radial spokes during flagellar beating^17,35^. However, several studies showed that mutant *Chlamydomonas* flagella lacking central pair and/or radial spokes began beating under specific conditions, such as high pressure, mechanical perturbation and very low ATP concentration, suggesting that the beating of flagella does not necessarily require the presence of central pair/radial spoke in the axoneme^24,36,37^. Thus, force oscillation by dynein on the doublet and the beating of flagella do not require a central pair.

The forces of “Single doublets” were much smaller than those of “Axonemes” and “Doublet bundles” and the predominant force was ~ 5 pN independent of the lengths of the microtubules (Fig. 2d). The reason for the small force and length-independency would be related to the structure of “Single doublets”. The “Single doublets” take curved and twisted form after disintegrated from “Axonemes”^18,38^. This results in short interaction lengths with straight polymerized microtubules. The interaction length at ~ 5 pN was ~ 390 nm (5 pN/12.7 pN μm^−1^), which was calculated by assuming that the mean force is proportional to the length of the microtubule interacting with “Single doublets” and the proportional coefficient is the same as that of “Axonemes”. The existence of predominant force indicates that the curvature and twisting angle are independent of the length of most “Single doublets”.

The fraction of force-generating dynein to total dynein molecules was calculated as follows. The mean force per dynein molecule on the doublet microtubule was calculated to be 0.11 pN from the force per length (12.7 pN μm^−1^) divided by the number of dyneins per micrometer (115 μm^−1^). The stall force of a single molecule of axonemal dynein was measured to be ~ 5 pN^25^. Assuming that the force generated by dynein molecules in dynein ensembles on the doublet is uniformly distributed from zero to the maximum force (~ 5 pN), similar to the force distributions of myosin during isometric contraction of muscle^39^, the mean force of dynein interacting with microtubules was half (2.5 pN) of the maximum force. The fraction of force-generating dynein was calculated to be 4.4% (0.11 pN/2.5 pN). This value is close to that (3%) calculated from the torque generated by the living bull sperm and the number of dynein molecules in the flagella^40^. Our results suggest that a small fraction of dynein on the doublet interacts simultaneously with microtubules.

### Duty ratio of purified dynein measured in the in vitro assay

To confirm the possibility of a small fraction of force-generating dynein, we measured the duty ratio of purified outer arm 21S dynein in the in vitro assay. The duty ratio was calculated to be 6.9% from the relationship between the dynein density on the glass surface and the velocities of microtubules (Fig. 3a). The low duty ratio was consistent with previous reports of outer arm dynein (1%, *Tetrahymena*; 8.1%, *Chlamydomonas*)^41,42^. The duty ratios of inner-arm dynein-c and -f of *Chlamydomonas* were measured to be 14% and 63%, respectively^30,43^. Kotani and colleagues showed that the sliding velocities of microtubules driven by mixtures of dynein-f and -c (~ 3 μm s^−1^ at a mixing ratio of 0.5) are between the velocities of dynein-f (1.2 μm s^−1^) and dynein-c (8 μm s^−1^); the researchers suggested that the dissociation of slow dynein-f from doublet microtubules is accelerated by fast dynein-c, reducing the duty ratio of dynein-f at a fast sliding velocity^43^. These results suggest that small fractions of outer and inner arm dynein molecules contribute simultaneously to the force generation and sliding of doublets.

To understand the reason for the low duty ratio in view of the ATPase cycle, the affinity of 21S dynein to microtubules was measured at various chemical states (Fig. 3b,c). The affinities to microtubules in the MD-AMPPNP, MD-ADP, and MD-apo states were ~ 30 times lower than the affinity at 1 mM ATP. The low affinities were consistent with the findings from previous qualitative work (Fig. 3b,c)^44^. The low affinity of 21S dynein to microtubules was in marked contrast to the strong binding states of cytoplasmic dynein under these nucleotide conditions^45^.

Microtubules interacting with dynein in the ADP-Vi state showed Brownian motion (Fig. 3c). The translational diffusion coefficient along microtubules calculated from Brownian motion was approximately 60 times smaller than that in the solvent (Fig. S1b). This result was consistent with the diffusion coefficient (50 times) obtained in previous work^44^. The low diffusion coefficient indicates that dynein in the ADP-Vi state weakly binds to microtubules through rapid association/dissociation^46^. Upon combining this result with that of MD-AMPPNP, MD-ADP, and MD-apo, it is concluded that the low affinities of dynein to microtubules in predominant states cause the low duty ratio of 21S dynein and the small fraction of force-generating dynein.

### Oscillatory movements of doublet microtubules

The molecular oscillation characteristics of the axoneme are major aspects of flagellar beating that must be understood. The axonemes prepared by the demembranation of flagella can beat similarly to live sperm flagella^2^. In this study, oscillation was observed in all preparations (Fig. 4). It should be noted that the highest frequency of oscillations (~ 16%) was measured from “Single doublets” compared to other dissected conditions (~ 4%). This would be related to the presence of radial spokes, which are present in “Axonemes” and “Doublet bundles” but are thought to be lost in “Single doublets”^11,18^. Radial spokes are reported to regulate dynein through mechanosignaling during flagellar beating^17^. They may also play a role in dynein regulation when axonemes have no bending. The relationship between the amplitude and frequency of oscillation was analyzed for the first time for “Doublet bundle” and “Single doublet” (Fig. 5). The present relationship of “Single doublet” (Fig. 5) was near the previous one (amplitudes of 4–40 nm and frequencies of 100–650 Hz) measured by approximately full-length axonemes bound on a glass surface^31,32,47^. These results suggest that the oscillation of the whole length of the axoneme originates from oscillating dynein molecules on “Single doublet”.

To understand the molecular characteristics of dynein during oscillation, we analyzed the force, step size and dwell time (Figs. 6, 7). The predominant length of the microtubule interacting with dynein presumably on curving and helical single doublet was calculated to be ~ 390 nm. The number of dynein molecules that generate force simultaneously was calculated to be ~ 2.0 (0.044 × 45) from the fraction (4.4%) and number of dynein molecules (115 × 0.39 = 45) attached to the 390-nm doublet. The estimated number of 1–3 molecules is consistent with the result that a few dynein molecules contribute to the oscillatory motion of a single doublet interacting with microtubules at an angle of 45–90°^18^. Therefore, a few dynein molecules among ~ 45 dynein molecules interacting with microtubules simultaneously generated oscillatory force observed in “Single doublet”.

Stepwise displacements of microtubules driven by dynein molecules on oscillatory force generation were detected for the first time on all preparations (Fig. 6). Step sizes and dwell times in stepwise displacement were analyzed by a step-finding algorithm^33^. The step size of 8-nm is related to the tubulin-dimer spacing of 8.2 nm in microtubules^28,45^. In addition to tubulin spacing, the power stroke of dynein should contribute to the step size. The power stroke distance (8.3 nm) of single-headed cytoplasmic dynein^45^ and translation distance (5–10 nm) of dynein in the axoneme^48,49^ are close to the observed step size and tubulin spacing in this study. Therefore, it is suggested that an ~ 8-nm step size is generated by the power stroke of dynein and/or tubulin-dimer spacing.

The dwell times in the ascending and descending phases and the profile of the power spectrum density provide information on the rough waveform of oscillation. The mean dwell times in both ascending and descending phases of oscillation were approximately equal among all preparations (Fig. 7d–i). This result suggests the possibility that the rough waveform in the ascending phase is similar to that in the descending phase. Most power spectrum densities have single main peaks and do not have clear peaks at twofold and threefold main-peak frequencies. These results are consistent with sinusoidal waves but not with asymmetric waveforms, such as sawtooth waveforms (Figs. 4, 6). This result is supported by the sinusoidal wave observed at the high frequency oscillation of the whole length of the axoneme^31,32,47^. How does the minus-end directed motor dynein generate sinusoidal wave? In the in vitro assay, we found that the translational diffusion coefficient along microtubules decreased to 1/60 in the presence of ADP-Vi compared to that in the solvent, indicating the presence of molecular friction (Fig. S1b). Molecular friction may play a role to suppress sudden drop in force. In summary, the similarity in these parameters (low duty ratio, ~ 8-nm step and roughly sinusoidal waveform) suggests that these essential properties of dynein are inherent to the molecule and independent of the axonemal architecture.

### Beating of flagella

The common mechanochemical properties of the three preparations on force generation and oscillatory motion contribute to the beating of sperm flagella. The mean frequencies (84 ± 51 Hz, mean ± s.d.) of all preparations shown in Fig. 5 were comparable to the beat frequencies of sea urchin flagella (46 Hz)^2^ and those of single doublets interacting with microtubules at certain angles^18^. The mean peak-to-peak amplitude of oscillation in the present work was 18 nm (Fig. 5), which corresponds to the sliding distances between microtubules. This result is shorter than the sliding distances (~ 100 nm) between adjacent doublet microtubules in flagella during the beating of sperm calculated from the curvatures of flagella^34,50^. This discrepancy is explained by the recent work as follows^51^. In the minimum axoneme model, purified outer arm dyneins are sandwiched between two microtubules that are crosslinked by DNA origami. One of the microtubules in the model is bound to the glass surface, which is similar to the arrangement in the present work. The dynein in this arrangement generates linear-oscillatory motion with a sliding distance of ~ 30 nm. The bending motion is induced in another arrangement in which one end of two microtubules is fixed and the other end moves free^51^, similar to the bending motion generated by local sliding in an axoneme^3^. The sliding distances between microtubules during the bending motion increase to 130 and 370 nm^51^. These results suggest that linear oscillation is converted to bending motion and that the linear-sliding distance between doublet microtubules increases when one end of doublet microtubules in the present work is fixed and the other end is allowed to move free. Therefore, we postulate that the common mechanochemical properties among the three axonemal substructures in the present work are the functional basis of flagellar beating.

## Materials and methods

### Dynein and microtubules

Tubulin and 21S dynein were purified from porcine brains and the sea urchin *Strongylocentrotus nudus*, respectively, as described previously^13^. Microtubules labeled with tetramethylrhodamine (TMR) and biotin (Sigma, B4639) were polymerized in the presence of 1 mM guanosine triphosphate (GTP) for 15–20 min at 37 °C. The molar ratio of unlabeled tubulin: TMR-labeled tubulin: biotinylated tubulin was 7:1:2. The avidinated beads bound firmly to the biotinylated microtubules. Polymerized microtubules were stabilized with 100 μM paclitaxel and stored at room temperature.

### Preparation of “Axonemes”, “Doublet bundles” and “Single doublets”

Three kinds of axonemal dissected conditions, that is, “Axonemes”, “Doublet bundles” and “Single doublets”, were used for force measurements. These structures were obtained by the following procedure. Fragmented axonemes (“Axonemes”) were prepared from sea urchin *Hemicentrotus pulcherrimus* sperm according to previous methods^23^. To prepare “Doublet bundles” and “Single doublets”, “Axonemes” were digested with elastase. All steps with elastase had to be conducted gently to prevent dynein fragmentation^19^. “Axonemes” were treated with reactivating solution (20 mM N-2-hydroxyethylpiperazine-N-2-ethane sulfonic acid (HEPES), 2 mM MgSO_4_, 2 mM Ethyleneglycol bis(2-Aminoethyl ether)-N,N,N,N tetraacetic acid (EGTA), 50 mM K-acetate, 1 mM dithiothreitol (DTT), pH 8.0; without ATP) containing 5 μg mL^-1^ elastase (Type III, Sigma, E0127) and 5 μg mL^-1^ trypsin inhibitor (Sigma, T9003) for 90 s at 25 ± 1 °C. Elastase was inhibited by 10 mM phenylmethanesulfonylfluoride (PMSF; Thermo Scientific, 36978). “Doublet bundles” or “Single doublets” were obtained by the disintegration of “Axonemes” in the reactivating solution containing 1 mM ATP at pCa4 and 20 μM ATP without Ca^2+^, respectively. The remaining PMSF was washed out by perfusing the reactivating solution containing 1 mg mL^-1^ casein and 150 mM K-acetate.

### Force measurement by optical trapping

Force measurement experiments were conducted using a custom-made optical tweezer system, as described previously^45,51^. The flow chambers were composed of 24 × 32 mm and 18 × 18 mm cover glasses adhered with double-sided tape (10 μm in thickness). The larger cover glasses were cleaned by a plasma cleaner (PDC-32G, HARRICK PLASMA, Ithaca, NY) beforehand. “Axonemes” in reactivating solution without ATP were perfused into chambers to adhere to the glass surface. Mutant kinesin (point-mutated G235A with 490 amino acids) that maintained a strong binding state to microtubules due to the loss of ATPase activity was used as glue to bind beads to “Axonemes”; a large force of > 100 pN was measured. Avidinated polystyrene beads of 1 μm in diameter were coated with mutant kinesin by a previously reported method^45^. Assay buffer (reactivating solution containing 20 μg mL^-1^ catalase, 100 μg mL^-1^ glucose oxidase, 20 mM glucose, 1% (v/v) 2-mercaptoethanol, 10 μM paclitaxel and 1 mg mL^-1^ casein) containing kinesin-coated beads, 1 mM caged ATP (P^3^-1-(2-nitrophenyl) ethyl ester of ATP, Dojindo, 345-05503) and 20 units mL^-1^ hexokinase (Toyobo, HXK-311) were introduced to the chamber. Beads were brought into contact with “Axonemes” via immotile mutant kinesin. Force generation by dynein was induced by the photolysis of caged ATP by the repetitive flashes (4 or 8 times) of a UV laser (CryLas GmbH, 1Q-355-2) for 50 ms with an interval of 250 ms. For the force measurements of “Doublet bundles” and “Single doublets”, microtubules in assay buffer were perfused to interact with dynein molecules on “Doublet bundles” or “Single doublets” and incubated for 2 min. Unbound microtubules were washed out by perfusing assay buffer. Assay buffer was perfused into the chamber containing avidinated beads (1 μm or 200 nm in diameter) instead of kinesin-coated beads, 1 mM caged ATP and 20 units mL^-1^ hexokinase. The beads were manipulated to attach to microtubules that had interacted with “Doublet bundles” or “Single doublets”.

For “Doublet bundles”, microtubules attached to dynein rows on “Doublet bundles” without central pairs were used. Thin doublet bundles that did not contain central pairs were selected by the weaker intensity of fluorescence. The thinnest filaments with weak fluorescence intensities on the glass surface were appeared after the disintegration of “Axoneme” at low ATP concentration without Ca^2+^. Because the fluorescence intensity decreases on excitation, the first frames after switching the field of view in the video was used to measure the intensity. The intensity of “Axoneme” was 12.2 ± 1.4 times of that of the thinnest filaments; the photon counts of 43.5 ± 5.2 × 10^3^ (n = 12) and 3.6 ± 0.8 × 10^3^ (n = 5) (mean ± s.e.) per micrometer for “Axoneme” and thinnest filaments, respectively. It is reasonable that the thinnest filaments are “Single doublets” because the axoneme is composed of nine doublet microtubules, a central pair, radial spokes and accessory proteins, and thus the protein mass of the axoneme should be slightly more than ten times of the mass of the “Single doublet”. Caged ATP was photolyzed by UV pulses of 8 repetitions for 50 ms with intervals of 250 ms to maintain a high ATP concentration for “Doublet bundles” and mainly single pulses with a duration of 50 ms for “Single doublets”. The full width at half maximum of UV intensity was ~ 23 μm.

The displacement of the beads was detected with a quadrant photodiode connected to a PowerLab system (ADInstruments) as described in a previous work^52^. The roll-off frequency of the amplifier for detecting the displacement was 30 kHz (or a time constant of 5 µs) and that of the force measurements was > 4 kHz (a time constant of < 40 µs). Therefore, the response time of the measurements was better than 40 µs. The fluorescent images of beads, microtubules and three dissected samples were recorded with an electron multiplying charge-coupled device (EMCCD) camera (iXon 897, Andor, South Windsor, CT). The displacement was calculated from the displacement of the bead multiplied by the attenuation factor (1.0–1.5)^53^. The experiment was performed at a temperature of 25 ± 1 °C.

Among all tracings of the displacements, we distinguished oscillation from oscillation-like responses by analysis with the following four requirements: (1) a power spectrum density at the major peak was more than twice as large as that at the minor peak, (2) oscillation continues for more than 4 cycles, (3) the peak-to-peak amplitude of a couple of waves is larger than 8 nm and (4) the oscillation starts within 2 s after the last UV flash. The first requirement was taken to obtain clear oscillation, the second and third requirements were taken to distinguish oscillation from noise, and the fourth requirements were taken to measure oscillation at high ATP concentrations because it decreased gradually by hexokinase activity and diffusing ATP molecules. The step size and the dwell time values were analyzed by a customized step-finding algorithm on MATLAB^33,52^. We adopted the highest peak of the S-value plot to acquire the best fit. We chose only the oscillations with clear peaks in the S-value plot of the step-finding algorithm.

### In vitro motility assay

Flow chambers that were ~ 3 μL in volume with 2 channels were composed of 24 × 32 mm and 18 × 18 mm cover glasses adhered with 3 slips of double-sided tape with 30 μm thicknesses as spacers^45^. The larger cover glasses were cleaned by a plasma cleaner beforehand. After pretreatment by the reactivating solution with 0.05 mg mL^−1^ casein and without ATP, 2.8–220 nM dynein was perfused into a chamber 2 times with an interval of 2 min. To obtain the low surface dynein density, 2.8 nM dynein was perfused once. After washing out excess dynein by perfusing 0.05 mg mL^−1^ casein, assay buffer containing 0.2% (w/v) methylcellulose (4000 cP, Sigma, M0512), 1 mM ATP and microtubules were perfused. The sliding movements of microtubules by dynein were observed under an epifluorescence microscope equipped with an objective lens (60×, Olympus, UPlan Apo) and a scientific complementary metal oxide semiconductor (sCMOS) camera (Andor Zyla). Twenty-five views were observed sequentially using a motorized stage (BIOS-105T, SIGMA KOKI). The positions of bright spots in microtubules translocated by dynein were determined by fitting their intensity profiles to two-dimensional Gaussian functions (Mark2. Kindly provided by Ken’ya Furuta, NICT). The sliding velocity was calculated from the displacements of microtubules that moved more than their lengths or for more than 8 s.

For the affinity experiment of 21S dynein to microtubules, 200 nM dynein was perfused twice in the chamber coated with 0.05 mg mL^−1^ casein. After washing out excess dynein, assay buffer was perfused containing microtubules with nucleotide (1 mM ATP, 1 mM AMPPNP, 1 mM ADP, or 1 mM ADP + 200 μM vanadate) or without nucleotide. After incubation for more than 3 min, the number of microtubules interacting with dynein was observed. When a microtubule bound to the glass surface for more than 5 frames (1 s), it was determined to be attached.

The concentration and the density of dynein attached to the chamber were measured by the following method: 3 μL of 55 or 220 nM dynein in the reactivating solution was perfused into a casein-coated chamber. Then, 3 μL of the reactivating solution was perfused in one side of the chamber, and overflowed solution containing unbound dynein on the glass surface was sucked from the other side. These procedures were repeated twice. The dynein concentrations in the sucked solutions were measured by the Bradford method. When perfusing 55 nM dynein, the percentages of unbound dynein were 11% and 19% in the 1st and 2nd sucked solutions, respectively. When perfusing 220 nM dynein, the values were 7% and 44% in the 1st and 2nd sucked solutions, respectively. It was assumed that all dyneins contained in solutions (3μL) attach to the upper and lower glass surfaces of the perfusion chamber and the attached dynein densities were calculated from area of the chamber, perfused volume, perfused dynein concentrations and ratios of dynein attached to the chamber.

### Statistical analysis

Statistical tests were performed with a library of statistical software tools (Kaleida Graph 4.5 and Excel). The step size and the dwell time values on the displacement traces in Figs. 6 and 7 were analyzed by a customized step-finding algorithm on MATLAB. The algorithm was originally developed by Kerssemakers et al.^33^. The error bars in figures indicate the standard error (s.e.). We used s.e. because the variance of the data we obtained was large and the phenomena we observed were considered to be stochastic.

## Supplementary Information

Supplementary Information.

## Data Availability

The datasets used and/or analyzed during the current study available from the corresponding author on reasonable request.
