# Supplementary material for: Versatile properties of dynein molecules underlying regulation in flagellar oscillation
Source: Sci Rep. 2023 Jun 29;13:10514. doi: 10.1038/s41598-023-37242-6 (PMC10310797; doi:10.1038/s41598-023-37242-6)
Supplement: Supplementary file 1 — Supplementary Information. [file 41598_2023_37242_MOESM1_ESM.pdf]

Supplemental information

Versatile properties of dynein molecules underlying regulation in flagellar oscillation

Takashi Fujiwara, Chikako Shingyoji and Hideo Higuchi

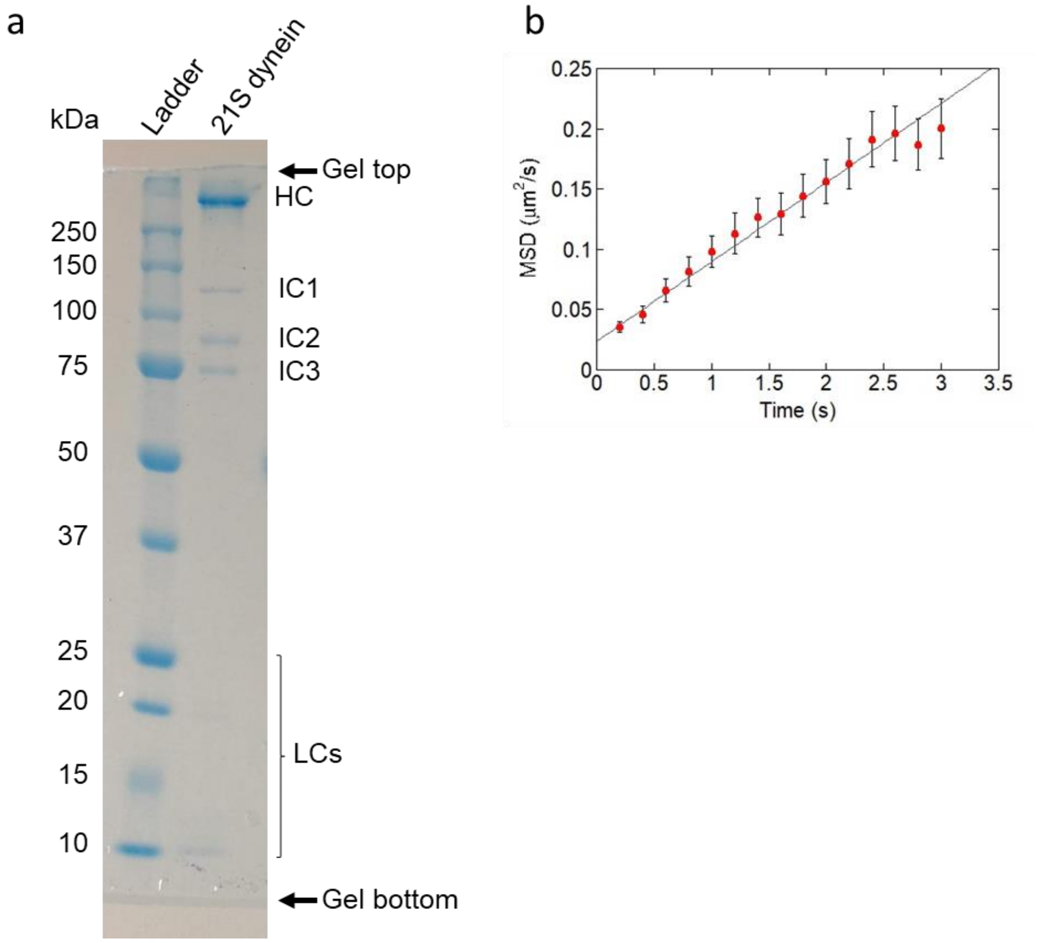

Fig. S1. (**a**) SDS-PAGE (5-20%) patterns of 21S dynein purified from sperm of the sea urchin *Strongylocentrotus nudus* showing the composition of heavy chain (HC), intermediate chains 1-3 (LC1-3) and light chains (LCs). (**b**) Mean Square distance (MSD) at ADP-Vi state. The translational diffusion coefficient, D, was calculated to be 3.3×10^4 nm^2 s^{-1} from the equation of MSD=2Dt. The viscosity coefficient, γ, was calculated to be 1.2×10^{-4} pNs nm^{-1} from $\gamma = k_B T/D$, where k_B and T are Boltzmann coefficient and absolute temperature.
